# Characterization of Eyeball Loss in Four Cities of Colombia

**DOI:** 10.7759/cureus.1677

**Published:** 2017-09-11

**Authors:** F. Hernán Moreno-Caviedes, Nórida Velez Cuellar, Margarita Caicedo Zapata, Gabriel Triana Reina, Azucena Sánchez

**Affiliations:** 1 School of Optometry, Fundación Universitaria Del Área Andina (colombia); 2 Epidemiology, Universidad El Bosque (colombia); 3 Universidad De La Salle (colombia), Fellow Asoprot

**Keywords:** anophthalmos, eye injuries, eye evisceration, ocular prosthesis

## Abstract

Purpose: Describe the socio-demographic characteristics of anophthalmic patients examined at specialized centers of four cities in Colombia to know the different causes of eyeball loss.

Method: A transversal retrospective study was done of 511 medical records from the specialized practices of four cities in Colombia. Socio-demographic data of patients who were seen between January 2011 and December 2013 were compiled. SOFA Statistics software v1.4.6 was used for this analysis. An analysis throughout the measures of central tendency for numerical variables was developed, and the descriptive statistics were used for the categorical variables.

Results: Almost 63% of the data belonged to male patients. Eyeball loss was more frequent in patients over 40 years of age. Fifty-one percent of the patients suffered eyeball loss due to traumatic causes, 40.2% due to pathological causes, and 4.6% due to congenital anomalies. The most frequent specific causes were glaucoma (19%), ocular cancer (15.4%), and home accidents (11,2%). Around 60% of the anophthalmic patients belonged to low socioeconomic level.

Conclusions: It is important to highlight that more than half of the analyzed anophthalmia cases originated in some type of trauma; this means that they could be considered potentially avoidable losses. Complications deriving from glaucoma became the most frequent cause of anophthalmia in the pathological origin group, which suggests a reflection regarding the strategies of early detection of the disease and access to proper treatment. It is also showed the need to develop an efficient system to manage information.

## Introduction

Anophthalmia or the absence of ocular structures inside the ocular socket may be of congenital origin, it can occur in an isolated way or as part of a chromosomal syndrome, and it may occur in an acquired form because of multiple situations [1].

The prevalence of births with anophthalmia in the general population has been estimated in 3 X 106 inhabitants, commonly with a bilateral presentation of the disease [2-3]. Alternately, a study in 212,479 consecutive live births found a prevalence rate of anophthalmia of 0.23% per 10,000 live births [4]. Some of the risk factors described for this condition include hereditary factors, environmental factors, maternal and gestational variables (multiple births, low weight at birth, and drug use), the own characteristics of the patient (over 40 years of age), and genetic alterations (chromosomal abnormalities, duplication syndrome, Wolf Hirschhorn syndrome, and triploidy), among others  [1,5].

Researchers analyzed the records of around 5.7 million births and reported rates of microphthalmia and anophthalmia at a rate of 0.92 to 2.29 per 100,000 births in France, Sweden, and the United states [6]. In 2006, Forrester MB et al. determined a ratio of 3.21 per 100,000 births for microphthalmia and anophthalmia in a total of 298,994  registered births between 1986 and 2001 in Hawaii [7].

A report published in Colombia by the National Institute of Health showed that the ocular anomalies represented 2.51% of all genetic congenital alterations for 2013 [8]. In 2016, 7,153 cases of congenital defects were reported by SIVIGILA (public health monitoring system) of which 82 cases (1.1%) were related to sensorial defects [9]; however, such reports do not have information about specific ocular anomalies. According to data from the National Administrative Department of Statistics (DANE), in Colombia, there is a total of 348,620 people (17.2%) with a disability related to the eyes [10], but the type of disease suffered by the patient is not discriminated. 

There is a lack of information about the different circumstances that cause the loss of the visual organ and the characteristics of the patients that suffer this disease. Besides, there is no clear data about the rate of congenital anophthalmia in the Colombian population. The purpose of this paper is to describe the socio-demographic characteristics of anophthalmic patients evaluated in specialized centers of four cities of Colombia to know the different causes of eyeball loss and to promote interest in studying this special condition.

## Materials and methods

Study design

Retrospective transversal study

Data collection

Anophthalmia medical records were collected from specialized eye rehabilitation practices that had a certified professional in four cities of Colombia (Barranquilla, Cali, Ibagué, and Pereira). Data were reviewed between January 2011 and December 2013. Records with other types of ocular alterations, such as microphthalmia, macrophthalmia, or corneal leukoma, were excluded.

Analysis of results

For the numerical variables, an analysis throughout the measures of central tendency was developed and descriptive statistics were used for the categorical variables. SOFA Statistics software V1.4.6 was used for data analysis (www.sofastatistics.com). Recommendations made in the Strengthening the Reporting of Observational studies in Epidemiology (STROBE) initiative were considered [11].

## Results

**Figure 1:**
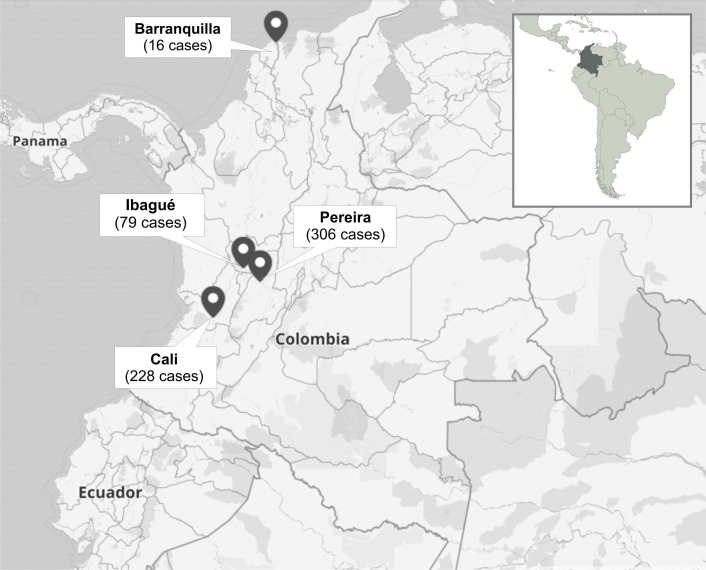
Distribution of cases of anophthalmia in four cities of Colombia

A total number of 629 cases of patients with the diagnosis of anophthalmia were analyzed in four cities: Barranquilla, Cali, Ibagué, and Pereira (Figure 1); 118 cases were excluded because they did not meet the inclusion criteria.

Around 63% (n=321) of the cases belonged to male patients, with an age range of six months to 90 years and an average age of 43.4 years for 455 cases that registered the patient´s age. Approximately 52% of cases were above 40 years’ old and around the fourth part of the data belonged to patients over 60 years’ old, which indicates a greater occurrence of anophthalmia as age increases, for the analyzed records (Table 1). Bilateral anophthalmia was described in 14 cases and the unilateral disease was presented without big differences between the right and left eyes.

**Table 1:** Demographic characteristics, affected eye, economic level, job type, and causes of anophthalmia in four cities of Colombia

	n	%
Gender		
Male	321	62.8
Female	190	37.2
Total	511	100
Age		
0-15	65	12.7
16-25	49	9.6
26-39	76	14.9
40-59	140	27.4
60-90	125	24.5
No data	56	11.0
Total	511	100
Affected eye		
Both eyes	14	2.7
Right eye	251	49.1
Left eye	246	48.1
Total	511	100
Socioeconomic status		
Low	147	29
Medium	89	17
High	4	1
No data	271	53
Total	511	100
Job type		
Student	40	7.8
Retired	34	6.7
Formal job	51	10.0
Informal work	129	25.2

 

The socioeconomic situation was known in 240 cases; 61.3% of the records belonged to patients of low socioeconomic level. Eyeball losses related to trauma were more frequent in men of low economic level. 

Forty-nine different types of jobs were identified in 257 cases that had such information; these were grouped into five categories where only 10% (n=51) of the cases reported to have formal employment. Regarding specific jobs, 10.8% of the cases were represented by housewives, followed by patients who said they were retired (6.7%), and others who worked in activities related to agriculture (4.3%). The cases of patients who were in elementary school or high school represented 6.1%. Other jobs included business people, drivers, administrative staff, technical staff, and mechanics, among others (Table 2).

**Table 2:** Frequency of anophthalmia according to specific occupation

Occupation	n	%
No data	229	44.8
Housewife	55	10.8
Others	54	10.6
Retired	34	6.7
Children under four years	25	4.9
Agriculture	22	4.3
Cleaning service	16	3.1
Primary school	16	3.1
High school student	15	2.9
Self-employed	13	2.5
Construction	11	2.2
College student	9	1.8
Salesman	6	1.2
Teacher	6	1.2
Total	511	100

**Figure 2:**
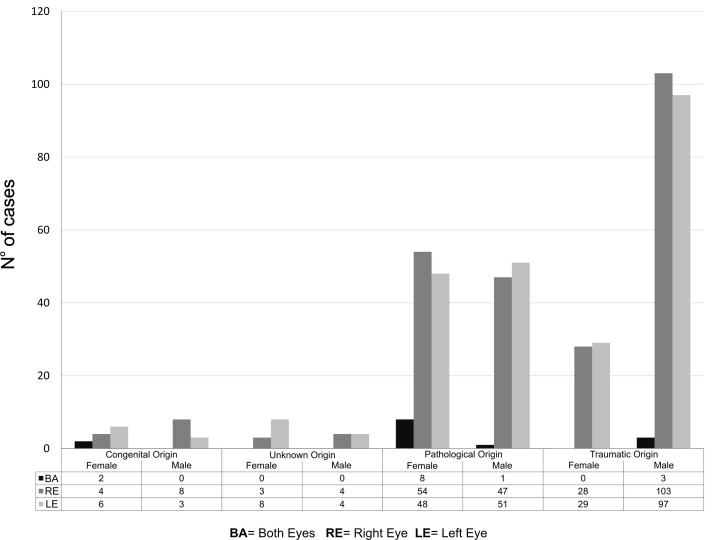
Description of the eyeball loss according to the type of cause, genre, and affected eye

The causes of the eye globe loss were grouped into four categories or etiological groups: traumatic, congenital, pathological, and unknown causes. Approximately 51% (n=260) of anophthalmia cases derived from some type of trauma. Eyeball loss related to common violence and home or traffic accidents were in a proportion of 4:1 for the male group versus the female group; patients between 40 and 59 years’ old presented a higher frequency of eye loss related to accidents and violence compared with the other groups. For the female group, eyeball loss due to pathological causes was the most common (Figure 2).

**Figure 3:**
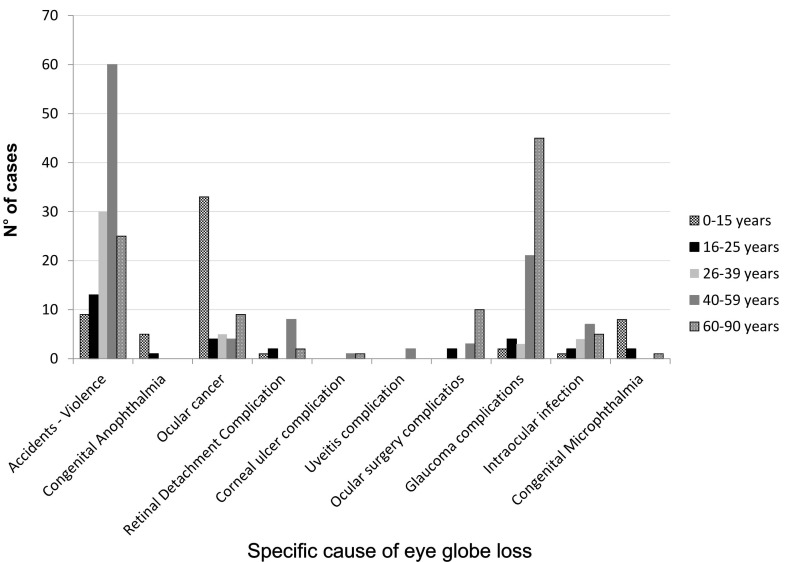
Distribution of eye globe loss according to specific cause and age

In the pathological causes group, the most frequent cause was glaucoma, followed by ocular cancer and intraocular infections. In patients over 60 years’ old, glaucoma was more frequent as well as secondary complications of ocular surgery, where cataract extraction with intraocular lens implant was the most common surgery. There was no information about the specific type of glaucoma. Retinoblastoma was the most common (73.7%) in the group with ocular cancer, in which patients under 15 years of age represented 55.9% of the cases. In addition, 23 cases of congenital anophthalmia were found (Figure 3).

Information about the use of an ocular prosthesis was obtained in 311 medical records; 30.5% (n=95) were already users of an ocular prosthesis by the time they were examined. Only 73 cases registered the time of use of an ocular prosthesis with an average time of 21.1 years and a maximum time of 55 years. Over 75% of the patients reported having used the same ocular prosthesis for more than 10 years.

In 85.9% of the cases, the type of surgery was known; evisceration was the most frequent (51.3%). Two cases of exenteration or the removal of the orbital contents and ocular adnexa were found.

There was neither information about the enucleations that were performed with or without an ocular implant, nor information about the types or characteristics of the implants used. Other procedures registered were enucleation (33.3%), dermal-fat graft (0.59%), and fornix reconstruction (0.39%). The medical records reviewed did not have relevant information about post-surgical follow-up or post-surgical complications.

## Discussion

There are a few studies about anophthalmia and eyeball loss in Colombia; however, this is the first paper that explores the characteristics of this anomaly in different cities of the country. The unilateral affection was presented without significant differences between the right and left eyes; the frequency for both was close to 50%. These results were similar to others authors in Brazil and the United States [7,12].

In 2013, Sousa RL et al. analyzed medical records in the western central area of Sao Paulo, and he observed that this anomaly affects a higher proportion of men over 40 years of age. Among the causes of anophthalmia, he found pathologies such as glaucoma (painful blind eye). His findings were consistent with the results of this paper, where complications related to glaucoma were 37.7% in the group of pathologies that caused eye loss. There are also consistencies regarding the age groups and the pathologies related to the eye globe loss. A greater occurrence of anophthalmia was related to traumatic incidents (work-related accidents, traffic accidents, and common violence) in male individuals, similar to that reported by other studies [13]. 

Incidents related to home accidents, common violence, traffic accidents, and work accidents made a total of 42.7% of the records analyzed, which indicates that about half of the anophthalmia cases could potentially be avoidable, just as is stated in other investigations [14-15].

In 2014, the Ministry of Health presented a report about the socio-economical determining factors of the violence in Colombia, and although it did not mention the frequency of victims with ocular damage, it did describe the distribution of different types of violence according to gender and geographical area [16]. The eye globe loss because of violence represented an important number in this investigation (17.3%); common violence events were the most frequent compared to domestic violence and armed conflict, having a higher occurrence among those who were between 26 and 59 years of age and with a proportion of 4:1 for the male genre, which is consistent with Ramos Souza et al., who found, in 2012, the highest rates of violence in populations between 15 and 49 years of age and with a higher frequency in the male gender [17].

More than half of the patients with the anophthalmia diagnosis were over 40 years of age, which might suggest that the possibility of suffering a condition that may lead to eyeball loss increases with age. Complications derived from glaucoma constituted the most frequent cause of loss of the organ of vision in the pathological origin group. This result is high with respect to other papers checked [18], which implies a reflection with respect to the strategies for the early detection of the disease in Colombia, the possibilities of access to proper treatment, and the treatment schemes currently used for the control of the disease.

The time for the replacement of the ocular prosthesis was extremely long since the average time of use of the same ocular prosthesis was more than 20 years, making evident the absence of a control and follow-up scheme for patients that have suffered the loss of one eye, considering that the use of a damaged ocular prosthesis might cause complications and chronic discomfort in the anophthalmic patient. This is similar to how it is described in studies done by specialists at the Cuban Institute of Ophthalmology in 2010 and 2014, where it was found that conjunctival fornix retraction, infectious processes, and allergic reactions are the most frequent conditions in patients who wear an ocular prosthesis [19-20]. This special type of patient requires professional support for the adequate care and maintenance of the artificial eye. Furthermore, a periodical replacement of the ocular prosthesis is necessary to avoid injuries and damage to the ocular socket tissue.

More than 60% of the cases analyzed belonged to the low economic levels; however, Colombia's health system does not cover the treatment expenses related to the ocular prosthesis. Perhaps it is necessary to review the current health policies to promote a truly comprehensive access to health services.

One of the limitations of this paper was the quality of the information available in many of the medical records checked since, in some cases, relevant data, such as the cause of the loss, education level, occupation, economic level, type of cause, and patient's age was not found. It is necessary to develop an adequate system for the management and collection of information. 

It is convenient to develop analytical studies that allow us to determine associations among the occurrence of anophthalmia and demographic, and clinical and etiological variables.

## Conclusions

There is a lack of information about the different circumstances that cause the loss of the eyeball and the characteristics of anophthalmic patients. Furthermore, there is no clear data about the rate of congenital anophthalmia in the Colombian population. In this study, over 40% of the records corresponded to patients in the productive age (between 26 and 60 years). The male genre was the most affected (63%), and ocular trauma was the main cause of eye loss. Eye losses related to trauma were more frequent in men of low economic level.

Glaucoma complications were the main pathological cause (37.7%) and retinoblastoma was the most frequent neoplasm in patients under 15 years of age. Most of the eye losses (68.3%) were related to potentially preventable events. Bilateral anophthalmia was found in 2.7% of the cases, and the unilateral compromise was similar for the right and left eyes.

Over 75% of the patients reported having used the same ocular prosthesis for more than 10 years, which indicates that in Colombia, there does not exist an adequate scheme for specialized control and follow-up to ocular prosthesis wearers. Moreover, 63% of records containing data about income belonged to the low economic strata. This is the first research that describes the causes of eyeball loss in different Colombian cities.
